# Baseline Cytomegalovirus Viremia at Cryptococcal Meningitis Diagnosis Is Associated With Long-term Increased Incident TB Disease and Mortality in a Prospective Cohort of Ugandan Adults With HIV

**DOI:** 10.1093/ofid/ofad449

**Published:** 2023-09-19

**Authors:** Jayne Ellis, Ananta S Bangdiwala, Caleb P Skipper, Lillian Tugume, Laura Nsangi, John Matovu, Katelyn A Pastick, Kenneth Ssebambulidde, Bozena M Morawski, Abdu K Musubire, Mark R Schleiss, David A J Moore, Joseph N Jarvis, David R Boulware, David B Meya, Barbara Castelnuovo

**Affiliations:** Infectious Diseases Institute, College of Health Sciences, Makerere University, Kampala, Uganda; Clinical Research Department, London School of Hygiene and Tropical Medicine, London, UK; University of Minnesota, Minneapolis, Minnesota, USA; University of Minnesota, Minneapolis, Minnesota, USA; Infectious Diseases Institute, College of Health Sciences, Makerere University, Kampala, Uganda; Infectious Diseases Institute, College of Health Sciences, Makerere University, Kampala, Uganda; Infectious Diseases Institute, College of Health Sciences, Makerere University, Kampala, Uganda; University of Minnesota, Minneapolis, Minnesota, USA; Infectious Diseases Institute, College of Health Sciences, Makerere University, Kampala, Uganda; University of Minnesota, Minneapolis, Minnesota, USA; Infectious Diseases Institute, College of Health Sciences, Makerere University, Kampala, Uganda; University of Minnesota, Minneapolis, Minnesota, USA; Clinical Research Department, London School of Hygiene and Tropical Medicine, London, UK; Clinical Research Department, London School of Hygiene and Tropical Medicine, London, UK; Botswana Harvard AIDS Institute Partnership, Gaborone, Botswana; University of Minnesota, Minneapolis, Minnesota, USA; Infectious Diseases Institute, College of Health Sciences, Makerere University, Kampala, Uganda; University of Minnesota, Minneapolis, Minnesota, USA; Infectious Diseases Institute, College of Health Sciences, Makerere University, Kampala, Uganda

**Keywords:** advanced HIV disease, *Cryptococcus*, cytomegalovirus, meningitis, tuberculosis

## Abstract

**Background:**

Adults with HIV-associated cryptococcal meningitis have overlapping burdens of cytomegalovirus (CMV) and tuberculosis (TB) coinfections. CMV infection/reactivation is strongly associated with CMV-specific memory T-cell activation and upregulation of type 1 interferons, which may lead to increased risk of TB disease and poor outcomes.

**Methods:**

We conducted a cohort study of 2-week survivors of cryptococcal meningitis during 2010–2021 to determine TB incidence and all-cause mortality over time stratified by baseline CMV status.

**Results:**

We followed 497 Ugandans with HIV-associated cryptococcal meningitis for a median (interquartile range) of 4.6 (2.6–53.9) months. Overall, 42% (210/497) developed incident TB disease or died. One-fifth (98/497, 19.7%) developed incident TB disease, and 29% (142/497) of participants died during follow-up. Of 259 participants with CMV viral load measured at baseline, 37% (96/259) had concurrent CMV viremia (defined as anyone with detectable CMV DNA in plasma/serum by qualitative polymerase chain reaction [PCR] detection). Of 59 with measured CMV immunoglobulin G (IgG), 100% had positive CMV IgG antibody serology (≥10 enzyme-linked immunosorbent assay units/mL). CMV viremia was positively associated with higher HIV viral load (196 667 vs 73 295 copies/mL; *P* = .002) and higher cerebrospinal fluid fungal burden (68 500 vs 14 000 cfu/mL; *P* = .002) compared with those without. Participants with high-level CMV viremia (defined as CMV viral load ≥1000 IU/mL) had twice the risk of incident TB (subdistribution adjusted hazard ratio [aHR], 2.18; 95% CI, 1.11–4.27) and death (aHR, 1.99; 95% CI, 1.14–3.49) compared with participants with no or low-level CMV viremia. There was no association between the CMV IgG index and the incidence of TB/death (*P* = .75).

**Conclusions:**

CMV viremia >1000 IU/mL at meningitis diagnosis was associated with increased incident TB disease and mortality during long-term follow-up. Future studies to determine the causal relationship and potential for therapeutic intervention are warranted.

## Graphical Abstract

**Graphical Abstract:**
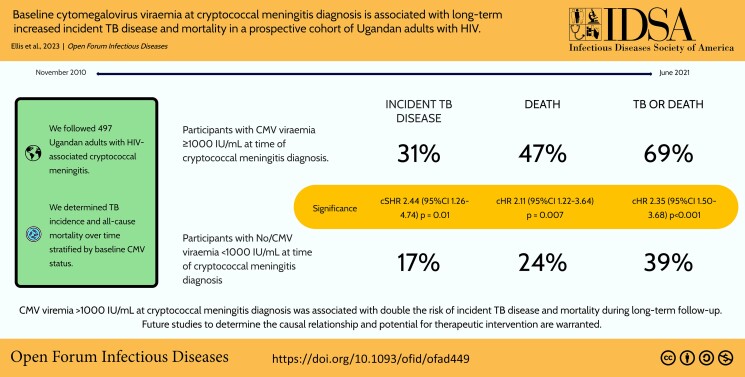
https://tidbitapp.io/tidbits/baseline-cytomegalovirus-viraemia-at-cryptococcal-meningitis-diagnosis-is-associated-with-long-term-increased-incident-tb-disease-and-mortality-in-a-prospective-cohort-of-ugandan-adults-living-with-hiv

*Cryptococcus* is the most common cause of HIV-associated meningitis globally, accounting for nearly 20% of all AIDS-related deaths [1]. Despite antifungal therapy, 10-week mortality in Sub-Saharan Africa remains between 24% and 45%, even in the context of clinical trials [2–5]. Acute deaths (≤2 weeks following diagnosis) among adults with HIV-associated cryptococcal meningitis are usually due to cryptococcosis; beyond 2 weeks, however, other HIV-related causes of death predominate [4], including other opportunistic infections. Due to advanced HIV disease, mortality continues well beyond hospital discharge, particularly in those with CD4 <50 cells/μL [6], and mortality at 12 months after cryptococcal meningitis diagnosis is between 40% and 78% in resource-limited settings [7, 8]. The key drivers of this persistently high mortality are unknown; however, recent data suggest that co-prevalent opportunistic infections including tuberculosis (TB) and cytomegalovirus (CMV) viremia may contribute to poor patient outcomes [9, 10].

It has been hypothesized that CMV-induced modulation of the host immune response (including CMV-specific memory T-cell activation and upregulation of antiviral type 1 interferons) results in negative feedback for type 2 interferons, such as interferon-gamma (IFN-γ), which are critical in the control of intracellular pathogens such as *Mycobacterium tuberculosis* and *Cryptococcus*, and that this downregulation may lead to an increased risk of reactivation of TB disease [11–14]. Supporting evidence comes from several studies. First, in a case–control study of Ugandan adults and children including 25 TB patients and 256 matched controls per case, the reported risk of TB disease was 3.4 times higher in participants with the highest CMV immunoglobulin G (IgG) levels (*P* = .007); CMV reactivation and/or re-infection events were proposed to be the driver for increased CMV IgG levels [12]. Second, in a prospective birth cohort study from South Africa, children acquiring CMV infection early in life were at higher risk of developing TB disease throughout childhood [13]. Third, a case–control study of 53 infants with TB and 205 matched controls found that CMV-specific CD8 T-cell activation was associated with an increased risk of developing TB disease and shorter time to TB diagnosis [14]. Taken together, these data may suggest that active CMV replication might modify risk of progression to active TB disease.

Adults with HIV-associated cryptococcal meningitis have high and overlapping burdens of CMV viremia (∼50%) and active TB disease (∼25%) coinfection [9, 10]. We sought to investigate whether CMV viremia and/or CMV IgG serostatus at the time of cryptococcal diagnosis were associated with incident TB disease and all-cause mortality over time among Ugandan adults with advanced HIV disease.

## METHODS

### Participants and Procedures

We conducted a cohort study of participants enrolled within 2 cryptococcal meningitis randomized controlled trials (RCTs) in Uganda to determine TB incidence and all-cause mortality over time stratified by baseline CMV viremia and CMV serostatus. The RCTs recruited consenting consecutive HIV-positive adults (≥18 years) with cryptococcal meningitis presenting to Mulago National Referral Hospital in Kampala or Mbarara Regional Referral Hospital. Cryptococcal Optimal ART Timing (COAT) Trial participants received intravenous amphotericin with adjunctive fluconazole and were randomized to either earlier antiretroviral therapy (ART) initiation at 1–2 weeks after diagnosis or deferred ART initiation at 4–6 weeks [15]. Adjunctive Sertraline for the Treatment of Cryptococcal Meningitis (ASTRO-CM) participants received standard cryptococcal meningitis treatment and were randomized to additionally receive adjunctive sertraline for 14 weeks, or matching placebo [3, 16]. Additional details may be found in the published study protocols [3, 15].

COAT and ASTRO trial follow-up was until 46 and 18 weeks after cryptococcal meningitis diagnosis, respectively. Thereafter, study participants were followed up at their local HIV clinic for ongoing care. For the purposes of this study, we included participants who survived at least 2 weeks after commencement of antifungal therapy, who were followed at the Infectious Diseases Institute (IDI), Kampala, Uganda, and had stored plasma or serum available for CMV testing.

### Data Collection

Baseline clinical and laboratory characteristics were collected as part of 1 of 2 RCTs. Longitudinal follow-up data following RCT termination at 18–46 weeks were extracted retrospectively from the electronic health records system (EHRS) at the IDI, where patients were followed up and seen regularly for medical care, with ART refills provided every 1–3 months.

### CMV Testing

All participants who had stored plasma or serum (collected during acute hospitalization) available for testing were included. DNA was extracted from 200 µL of plasma using the QIAcube HT (QIAGEN, Hilden, Germany) and QIAamp 96 Virus DNA QIAcube kits, according to the manufacturer's instructions. All samples were eluted in 100 μL of polymerase chain reaction (PCR)–grade water and were stored at −20°C. Multiplex quantitative PCR was performed using the LightCycler 480 System (Roche, Basel, Switzerland). Laboratory staff processed coded samples and were blinded to participant data and outcomes. CMV quantification of copies/mL values were converted to international units (IU) using the World Health Organization International Standard for Human CMV for Nucleic Acid Amplification Techniques (National Institute for Biological Standards and Control code 09/162). CMV viremia was defined as anyone with detectable CMV DNA in plasma or serum using the qualitative PCR detection thresholds defined by the test manufacturers.

CMV IgG serostatus was assessed using a CMV IgG enzyme-linked immunosorbent assay (ELISA; Diamedix, Miami Lakes, FL, USA), following the manufacturer's instructions. Specimens were run in triplicate at 1:100 dilution, with absorbance measured at 450 nm. Specimens with a measured ELISA unit level of ≥10 ELISA units/mL (EU/mL) were considered positive.

### TB Diagnoses

Participants were screened for tuberculosis symptoms using the 2010 World Health Organization questionnaire at each visit [17]. Those who answered “yes” to any of the 4 questions or who had any other clinical manifestations suggestive of tuberculosis underwent further TB screening. TB diagnostics were employed at physician discretion based on clinical syndrome. Incident TB disease was defined as microbiologically confirmed or clinical TB, diagnosed at any time during the study period.

### Ethics

#### Patient Consent

All participants (or a surrogate in cases of mental incapacity) provided written informed consent for inclusion in the RCT, including consent for sample storage for future testing [3, 15, 16].

Approvals for the 2 RCTS were obtained from the Mulago Institutional Review Board (COAT 2009-002, ASTRO 429), Uganda National Council for Science and Technology, and University of Minnesota. The use of data routinely collected at the IDI was approved by Makerere University Faculty of Medicine Research and Ethics committee (approval number: 120–2009). The requirement for verbal or written consent was waived as data were analyzed after stripping unique personal identifiers.

### Statistical Analyses

Baseline demographics and clinical characteristics were summarized as counts and percentages for categorical data, or medians (with interquartile ranges) and means (with ranges) for continuous data, depending on data distributions.

CMV viremia at the time of cryptococcal meningitis diagnosis was the primary exposure of interest; the proportion of participants with CMV viremia was determined and baseline variables were compared by CMV viremia coinfection status using the chi-square or Fisher exact test for proportions; the Mann-Whitney *U* test or Student *t* test was used for continuous data. To explore associations between CMV viral load (VL) and outcomes, we stratified those with evidence of CMV viremia into “low” and “high” CMV VL groups using a threshold of ≥1000 CMV IU/mL (a preemptive treatment threshold frequently used in hemopoietic stem cell transplant cohorts) [18]. We compared baseline characteristics and clinical outcomes across CMV status: (i) CMV viremia vs no CMV viremia and (ii) ≥1000 CMV IU/mL vs a composite exposure of no CMV viremia or CMV <1000 CMV IU/mL.

CMV IgG serostatus was the secondary exposure of interest. All study participants who had CMV serologic testing conducted were seropositive; therefore, we dichotomized participants into “low” and “high” CMV IgG index groups, using the 75% quartile range as the threshold (≥227 EU/mL), and compared outcomes across these 2 groups.

Incident TB and all-cause mortality during follow-up were the dependent variables of interest. Cumulative incident TB and mortality rates per 100 person-years (and their respective 95% CIs) were calculated. Incidence rate ratios (and 95% CIs) were calculated to compare outcomes stratified by baseline CMV viremia status and CMV serology status.

A survival regression model based on cumulative incidence function to calculate unadjusted and adjusted subdistribution hazard ratios (SHRs) was used to evaluate for an association between CMV viremia and incident TB given the competing risk of death. Univariable and multivariable Cox proportional hazards models were used to evaluate associations between baseline CMV viremia and (i) mortality and (ii) a composite end point of TB/mortality. The following baseline covariates were considered as potential confounders: age, sex, study, CD4 count, Glasgow comma score (GCS; <15), ART status, and TB preventative therapy (TPT) receipt. Study, sex, and CD4 count were included a priori; thereafter, using cross-tabulations, only covariates that were associated with both CMV viremia and incident TB or mortality were included in the regression models. HIV viral load, CSF white cell count (WCC), and CSF cryptococcal quantitative colony count (QCC) were considered to potentially be on the causal pathway and were therefore not subjected to adjustment. To assess for effect modification between CMV viremia and ART status, a likelihood ratio test was conducted. Using the same approaches, univariable and multivariable competing risk regression models and Cox proportional hazards models were constructed to look for any associations between CMV IgG index and incident TB and mortality. Cox proportional hazards models were also used to explore associations between other covariates and mortality, including CD4 count and ART status at baseline, and receipt of TPT.

Participants were right-censored at the date last known to be alive and TB-free; similarly, participants who were lost to follow-up and those who had their care transferred to another ART clinic were right-censored at the date they were last known to be alive. All analyses were performed in Stata, version 16 (StataCorp, College Station, TX, USA). *P* values have not been adjusted for multiple comparisons.

## RESULTS

**Figure 1.**
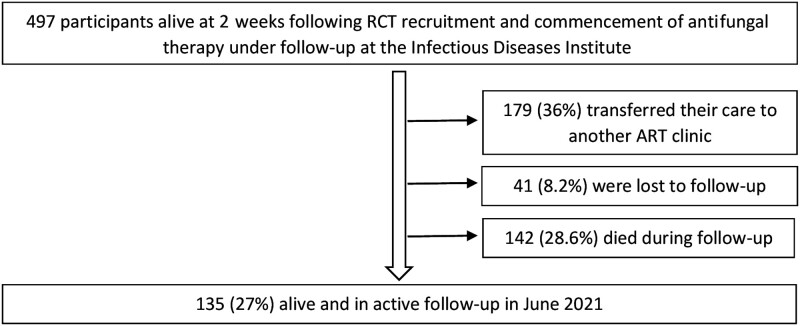
Follow-up status for 497 participants alive at 2 weeks following commencement of antifungal therapy with follow-up at the Infectious Diseases Institute. Abbreviation: ART, antiretroviral therapy.

Between November 2010 and May 2017, 637 adults with HIV-associated cryptococcal meningitis were recruited into the COAT (n = 177) and ASTRO (n = 460) RCTs; 497 participants (78%) survived at least 2 weeks after commencement of antifungal therapy and were subsequently followed up at the Infectious Diseases Institute clinic (Supplementary Table 1). Follow-up was for a median (interquartile range [IQR]) of 4.6 (2.6–53.9) months, for a total of 1159 person-years (Figure 1).

During the period November 2010–June 2021, 210/497 participants (42.3%) either developed TB or died. Twenty-nine percent (142/497) of participants died, with an overall cumulative mortality rate of 12.2/100 person-years (95% CI, 10.4–14.4/100) and a median time to death (IQR) of 137 (78–1616) days. Nearly one-fifth (98/497, 19.7%) developed incident TB disease during follow-up. Less than a quarter (116/497, 23%) of participants received any TB preventative therapy during the study period. On univariate Cox analyses, there was no association observed between baseline CD4 count category or ART status and death. Receipt of TB preventive therapy was, however, strongly associated with reduced hazard of death during the study period (crude hazard ratio [cHR], 0.01; 95% CI, 0.001–0.78; *P* < .001) (Supplementary Table 2).

The following analyses were restricted to include only participants who underwent baseline CMV VL testing. Of these 259 participants, 38% (98/259) were female, and the mean age (range) was 35 (18–60) years. All participants had advanced immunosuppression, with a median CD4 count (IQR) of 16 (7–52) cells/μL. At the time of cryptococcal meningitis diagnosis, 34% of participants were on ART (87/259) for a median duration (IQR) of 28 (5.8–154) weeks. Less than 20% (18.9%, 49/259) of participants reported any history of TB disease at the time of trial enrollment (Table 1). Baseline cryptococcal fungal QCC was higher in those with CMV viral load testing: however, there were no other significant differences in baseline characteristics between those who received CMV viral load testing and those who did not (Supplementary Table 1).

**Table 1.** Baseline Characteristics of 259 Adults With HIV-Associated Cryptococcal Meningitis With Stratification by (i) CMV Viremia Coinfection Status and (ii) CMV Serostatus

Variable	Total Cohort With CMV VL, No. (%) or Median (IQR)	No CMV Viremia, No. (%) or Median (IQR)	CMV Viremia, No. (%) or Median (IQR)	*P* Value^a^	Low CMV IgG Index, No. (%) or Median (IQR)	High CMV IgG Index, No. (%) or Median (IQR)	*P* Value^b^
Total	259	163 (62.9)	96 (37.1)	…	45 (76.3)	14 (23.7)	…
Male	161 (62.2)	101 (62.7)	60 (37.3)	…	31 (83.8)	6 (16.2)	…
Female	98 (37.8)	62 (63.3)	36 (36.7)	.93	14 (63.6)	8 (36.4)	.08
Age, y	…	…	…	…	…	…	…
≤30	83 (32.0)	49 (59.0)	34 (41.0)	…	12 (75.0)	4 (25.0)	…
31–50	168 (64.9)	108 (64.3)	60 (35.7)	…	32 (76.2)	10 (23.8)	…
>50	8 (3.1)	6 (75.0)	2 (25.0)	.56	1 (100)	0 (0)	1.00
ART	…	…	…	…	…	…	…
No	172 (66.4)	99 (57.6)	73 (42.4)	…	45 (76.3)	14 (23.7)	…
Yes	87 (33.6)	64 (73.6)	23 (26.4)	.01	…	…	…
Median time on ART, d	195 (41–1076)	165 (47–1028)	335 (35–1187)	.82	…	…	…
CD4 category, cells/μL	…	…	…	…	…	…	…
<50	191 (74.3)	114 (59.7)	77 (40.3)	…	41 (82.0)	9 (18.0)	…
50–99	46 (17.9)	33 (71.7)	13 (28.3)	…	1 (20.0)	4 (80.0)	…
≥100	20 (7.8)	15 (75.0)	35 (25.5)	.16	3 (75.0)	1 (25.5)	.001
Median HIV viral load, copies/mL	126 725 (8384–341 919)	73 295 (2919–245 107)	196 667 (24 554–387 068)	.002	237 610 (102 515–377 563)	405 292 (206 893–604 115)	.07
GCS < 15	74 (28.6)	44 (59.5)	30 (40.5)	…	10 (66.7)	5 (33.3)	…
GCS = 15	185 (71.4)	119 (64.3)	66 (35.6)	.46	35 (79.6)	9 (20.4)	.31
Median CSF WCC, cells/μL	4 (4–80)	4 (4–98)	4 (4–45)	.03	37.5 (4–110)	27.5 (4–45)	.71
Median CSF protein, mg/dL	60 (27–124)	60 (27–124)	60 (26–122)	.89	110 (44–179)	81 (72–182)	.66
Median CSF QCC, cfu/mL	35 000 (1260 –229 000)	14 000 (320–206 000)	68 500 (6850–349 000)	.002	214 000 (9000–403 000)	23 800 (320–218 000)	.07
History of TB	210 (81.1)	30 (61.2)	19 (38.8)	.78	12 (92.3)	1 (7.70)	.16
Receipt of TPT	49 (18.9)	46 (73.0)	17 (27.0)	.06	12 (80.0)	3 (20.0)	.70

For categorical variables, the *P* value is from the chi-square test if there were ≥5 participants in each category, or from the Fisher exact if there were <5 participants. For noncategorical variables, *P* values are from the Mann-Whitney *U* test throughout due to the nonparametric distribution of all data.

Abbreviations: ART, antiretroviral therapy; CMV, cytomegalovirus; CSF, cerebrospinal fluid; GCS, Glasgow Coma Scale; IgG, immunoglobulin G; QCC, quantitative cryptococcal culture; VL, viral load; WCC, white cell count.

a *P* value compares those without CMV viremia vs those with CMV viremia at the time of cryptococcal meningitis diagnosis.

b *P* value compares those with a low-level CMV IgG index vs those with a high-level CMV IgG index (≥227 enzyme-linked immunosorbent assay units/mL).

Thirty seven percent (96/259) had concurrent CMV viremia at the time of cryptococcal meningitis diagnosis. Among participants with CMV viremia, the median CMV VL (IQR) was 524 (152–2301) IU/mL; 14% (36/259) of participants had a CMV viral load ≥1000 IU/mL at baseline. Participants not receiving ART at the time of enrollment were more likely to have concurrent CMV viremia compared with participants receiving ART (42.4% vs 26.4%; *P* = .01). The median CSF fungal burden (IQR) was higher among those with CMV viremia (68 500 [6850–349 000] cfu/mL) compared with those without (14 000 [320–206 000] cfu/mL; *P* = .002). The median baseline HIV VL (IQR) was also higher among those with evidence of CMV viremia (196 667 [24 554–387 068] copies/mL) compared with those without (73 295 [2919–245 107] copies/mL; *P* = .002). Sex, age, CD4 count, TB history, and TPT receipt were similar in participants with and without CMV viremia. Similar between-group differences were observed when the ≥1000 CMV IU/mL group was compared with the composite group without CMV or with CMV <1000 CMV IU/mL (Supplementary Table 3).

All participants with CMV serology results available (n = 59) had positive serologies, with a median CMV ELISA result (IQR) of 156 (128–227) EU/mL; 14 participants (23.7%) had a “high” CMV IgG index (>227 EU/mL). The proportion of participants in the different CD4 count categories differed in participants with a “high” CMV IgG index and those without (*P* = .01). Other baseline covariates were similar in participants with high and low CMV serology indices (Table 1).

The cumulative incidence mortality rate was 80% higher among participants with CMV viremia (16.2 deaths/100 person-years; 95% CI, 11.6–22.6) compared with those without CMV viremia (8.72 deaths/100 person-years; 95% CI, 6.31–12.0), with a cumulative mortality incidence rate ratio of 1.86 (95% CI, 1.13–3.04). Among those with CMV VL ≥1000 IU/mL, the cumulative incidence mortality rate was 23.2 deaths per 100 person-years (95% CI, 14.4–37.4), equating to a cumulative mortality incidence rate ratio of 2.41 (95% CI, 1.31–4.23; *P* = .002) when compared with the composite group of participants without CMV viremia or with CMV viremia <1000 IU/mL. Similar associations were observed with incident TB/death as the outcome of interest; among those with CMV VL ≥1000 copies/mL, the cumulative TB/death rate per 100 person-years was 83.2 (95% CI, 56.2–123.2), with a cumulative TB/mortality incidence rate ratio of 4.19 (95% CI, 2.57–6.61/95% CI, 2.51–6.73) compared with the composite group of participants without CMV viremia or with CMV viremia <1000 IU/mL at baseline (*P* < .001) (Table 2).

**Table 2.** Cumulative Incidence Mortality Rates and Ratios for Mortality and TB Disease by Baseline CMV Viremia Status and CMV IgG Index

Variable	No.	No. of Deaths	Person Time, y	Cumulative Mortality Rate/100 Person-Years (95% CI)	Cumulative Mortality Incidence Rate Ratio (95% CI)
Participants with CMV VL data	259	71	634	11.2 (8.87–14.1)	…
No CMV viremia	163	37	424	8.72 (6.31–12.0)	1
CMV viremia	96	34	210	16.2 (11.6–22.6)	1.86 (1.13–3.04)
No/CMV viremia, <1000 IU/mL	223	54	561	9.6 (7.4–12.6)	1
CMV viremia ≥1000 IU/mL	36	17	73	23.2 (14.4–37.4)	2.41 (1.31–4.23)
Participants with CMV serology data	59	21	263	7.98 (5.20–12.2)	…
Low CMV serology index (<227 EU/mL)	45	18	192	9.4 (5.9–14.9)	1
CMV serology index ≥227 EU/mL	14	3	71	4.2 (1.4–13.11)	0.45 (0.08–1.55)
		No. of TB Cases or Deaths		Cumulative TB or Death Rate/100 Person-Years (95% CI)	Cumulative TB or Death Incidence Rate Ratio (95% CI)
Participants with CMV VL data	259	111	463	24.0 (19.9–28.9)	…
No CMV viremia	163	63	316	19.9 (15.6–25.5)	1
CMV viremia	96	48	147	32.6 (24.6–43.3)	1.63 (1.10–2.42)
No/CMV viremia (<1000 IU/mL)	223	86	433	19.8 (16.1–24.5)	1
CMV viremia ≥1000 IU/mL	36	25	30	83.2 (56.2–123.2)	4.19 (2.57–6.61)
Participants with CMV serology data	69	42	183	22.9 (17.0–31.0)	…
Low CMV serology index (<227 EU/mL)	45	26	139	18.7 (12.7–27.4)	1
CMV serology index ≥227 EU/mL	14	7	44	16.1 (7.67–33.7)	0.86 (0.31–2.04)

Abbreviations: CMV, cytomegalovirus; EU/mL, enzyme-linked immunosorbent assay units/mL.

**Figure 2.**
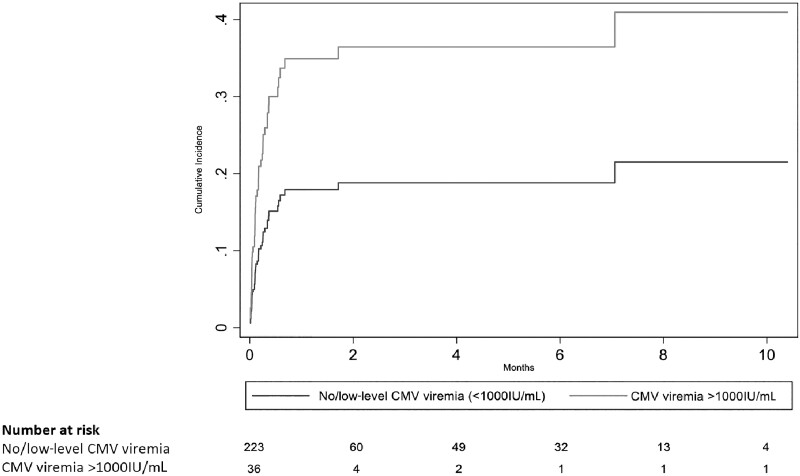
Cumulative incidence of active TB disease during follow-up among 259 patients diagnosed with HIV-associated cryptococcal meningitis, stratified by CMV viral load, using a competing risk regression model. Abbreviation: CMV, cytomegalovirus.

With adjustment for sex, study, CD4 count, and ART status, and accounting for the competing risk of death, participants with CMV VL ≥1000 IU/mL had more than twice the risk of incident TB disease during follow-up compared with participants without CMV viremia or with CMV viremia <1000 IU/mL at baseline (subdistribution aHR, 2.16; 95% CI, 1.10–4.23) (Figure 2). Similarly, using multivariable Cox analysis, with adjustment for sex, study, CD4 count, and ART status, participants with CMV VL ≥1000 IU/mL had twice the risk of death during follow-up compared with participants without CMV viremia or with CMV viremia <1000 IU/mL at baseline (aHR, 1.99; 95% CI, 1.14–3.49). The hazard of TB/death was >2 times as high for those with CMV VL ≥1000 IU/mL as to compared with participants without CMV viremia or with CMV viremia <1000 IU/mL at baseline (aHR, 2.14; 95% CI, 1.35–3.38) (Table 3). There was no evidence of effect modification between CMV viremia and ART status (*P ≥* .05 for all dependent variables). There was no association observed between CMV IgG index (“low” vs “high”) and incidence of TB or death (Tables 2 and 3).

**Table 3.** Mortality and Incident TB Disease by Baseline CMV Viremia Status and CMV Serology Index, Multivariate Analysis Using Survival Regression Modeling

Variable	No.	No. of TB Cases	CSHR For TB (95% CI)	*P* Value	ASHR For TB^a^ (95% CI)	*P* Value
Total cohort	497	98	…		…	…
No CMV viremia	163	31	1		1	…
CMV viremia	96	19	1.17 (0.65–2.10)	.61	0.92 (0.49–1.73)	.79
No/CMV viremia (<1000 IU/mL)	223	39	1		1	…
CMV viremia ≥1000 IU/mL	36	11	2.44 (1.26–4.74)	.01	2.18 (1.11–4.27)	.02
Low CMV IgG Index (<227 EU/mL)	45	10	1		1	…
CMV IgG Index ≥227 EU/mL	14	4	1.28 (0.41–4.03)	.67	1.85 (0.59–5.83)	.29
** **	No.	No. of Deaths	cHR for Mortality (95% CI)	*P* Value	aHR for Mortality^a^ (95% CI)	*P* Value^b^
Total cohort	497	142	…		…	…
No CMV viremia	163	37	1		1	…
CMV viremia	96	34	1.65 (1.03–2.62)	.03	1.56 (0.97–2.53)	.07
No/CMV viremia (<1000 IU/mL)	223	54	1		1	…
CMV viremia ≥1000 IU/mL	36	17	2.11 (1.22–3.64)	.007	1.99 (1.14–3.49)	.02
Low CMV IgG Index (<227 EU/mL)	45	18	1		1	…
CMV IgG index ≥227 EU/mL	14	3	0.49 (0.14–1.67)	.25	0.54 (0.15–1.94)	.32
		No. of TB Cases or Deaths	cHR for TB or Death (95% CI)	*P* Value	aHR for TB or Death^a^ (95% CI)	*P* Value^b^
Total cohort	497	210	…		…	…
No CMV viremia	163	63	1		1	…
CMV viremia	96	48	1.43 (0.98–2.08)	.06	1.24 (0.84–1.83)	.28
No/CMV viremia (<1000 IU/mL)	223	86	1		1	…
CMV viremia ≥1000 IU/mL	36	25	2.35 (1.50–3.68)	<.001	2.14 (1.35–3.38)	.002
Low CMV IgG Index (<227 EU/mL)	45	26	1		1	…
CMV IgG index ≥227 EU/mL	14	7	0.86 (0.37–1.97)	.71	1.03 (0.43–2.48)	.94

Abbreviations: aHR, adjusted hazard ratio; aSHR, adjusted subdistribution hazard ratio; CHR, crude hazard ratio; CMV, cytomegalovirus; cSHR, crude subdistribution hazard ratio; EU/mL, enzyme-linked immunosorbent assay units/mL.

a Adjustment for sex, study, ART status (categorical variables), and CD4 count (continuous linear variable).

b *P* value from likelihood ratio test.

## DISCUSSION

In our cohort of 497 adults, clinical outcomes following treatment for cryptococcal meningitis were poor, with >40% of participants either developing TB (20%) or dying (29%) during follow-up. CMV viremia ≥1000 IU/mL at cryptococcal meningitis diagnosis was strongly and independently associated with a >2-fold increased risk of incident TB disease or mortality during follow-up. There was a trend toward increased TB/death among participants with any CMV viremia (including those with CMV viremia <1000 IU/mL), but this trend did not meet statistical significance, suggesting that only CMV viremia ≥1000 IU/mL was of prognostic significance within this cohort. Although we cannot draw any causal inference from our study, if the relationship between CMV viremia and TB/death was causal, CMV viremia ≥1000 IU/mL represents a potentially modifiable risk factor to improve long-term TB and survival outcomes in adults with advanced HIV disease and cryptococcal meningitis.

CMV viremia has repeatedly been shown to be an independent risk factor for progression to both AIDS and death among persons with HIV [19–25]. In agreement with earlier published data from our group in Uganda [9], these data demonstrate a strong positive association between CMV viremia and all-cause mortality, and there is now increasing interest in CMV/TB co-pathogenicity based on both epidemiological and immunological data [11–14]. Compelling population-level evidence for an interaction between CMV and TB comes from a birth cohort of 963 South African infants who were serially tested for CMV and prospectively screened for *Mycobacterium tuberculosis* infection during childhood [13]. Infants who acquired CMV infection in the first year of life had a 3-fold increased risk of incident TB when compared with those who had not acquired CMV, with a consistent biological gradient showing that children with a high cytomegalovirus load were at greatest risk of tuberculosis disease [13]. These data suggest that the high tuberculosis progression rates observed in childhood may be partially mediated by CMV acquisition and immunomodulation. Our data further support the hypothesis of a significant interaction between CMV and TB, which may contribute to the high TB progression rates observed in advanced HIV disease.

CMV is a ubiquitous herpes virus, and in most low- and middle-income countries, where TB burden is highest, universal CMV seroconversion is seen in the first few years of life. In a Ugandan study, 95% of participants had immunological evidence of CMV infection by 5 years of age [26]. Following acute infection, CMV can sustain lifelong latency within the host with potential for intermittent reactivations. Due to advanced immunosuppression, CMV reactivation and/or reinfection episodes are common in advanced HIV disease [27], and up to 50% of adults with CD4 cell counts ≤100 cells/µL have detectable CMV viremia [9, 24]. Although low-level CMV viremia is often asymptomatic, CMV is highly immunogenic and is associated with profound immune activation including T-cell differentiation and upregulation of type 1 interferons (T1IFN) [11].

T1IFNs have antiviral activity (whereby these cytokines stimulate the production of innate antiviral proteins and promote effector CD8+ T-cell responses), but in parallel, type 1 T helper cell (Th_1_) IFN-γ responses for intracellular pathogens, such as *Cryptococcus* and *Mycobacterium tuberculosis*, are downregulated [28]. There is increasing recognition with data from several studies of the pathogenic role that T1IFNs play in TB and other bacterial infections due to downstream antagonism of IFN-γ and interleukin-1 [28]. In a South African cohort, patients with active TB disease demonstrated a blood-based transcriptome dominated by a T1IFN-inducible gene signature that correlated with the extent of radiographic lung disease, and the transcriptome diminished with successful treatment, suggesting that overexpression of T1IFN may contribute to TB disease pathogenesis [29]. Second, overexpression of T1IFN response genes have also been detected early in TB contacts who progressed to active disease, indicating that peripheral activation of T1IFNs precedes the onset of active TB disease [29–32]. While more mechanistic studies are required to characterize CMV-induced immunomodulation in advanced HIV disease, we hypothesize that CMV-induced T1IFN-mediated immunomodulation may in part underpin the epidemiological and clinical interaction between CMV and TB observed.

Our study has several limitations. First, not all participants received both CMV VL and CMV serology testing; however, other than baseline cryptococcal fungal burden being higher in those with CMV viral load testing, no other significant differences existed between those who did and those who did not receive CMV testing. The small sample size may have led to a type 2 error with respect to the lack of observed association between CMV IgG index and outcome, insofar as there was a nonsignificant trend toward increased incident TB in those with higher CMV IgG indices. Second, longitudinal follow-up data including incident TB diagnoses were extracted retrospectively from the IDI EHRS, and we recognize the limitations inherent to this. Additionally, participants were not systematically screened for active TB disease at each clinic visit, and therefore there is a notable risk of TB outcome underascertainment in this high-risk population. However, we do not anticipate that this potential for outcome misclassification would have been differential across CMV exposure status. Third, 41 participants (8%) were lost to follow-up, and 36% (179/497) of participants transferred their care to another ART clinic during the study period.

In summary, our data further highlight that coinfections are common among adults with cryptococcal meningitis and systematic screening for opportunistic infections is warranted. TB remains the single biggest killer among people with advanced HIV disease, but our understanding of who progresses to active TB and when is incomplete. We report for the first time that CMV viremia ≥1000 IU/mL is associated with double the risk of incident TB disease and mortality among adults with HIV-associated cryptococcal meningitis. These data add to the growing body of evidence that targeted anti-CMV therapy may be a potential therapeutic intervention to improve TB and long-term survival outcomes in advanced HIV disease and cryptococcal meningitis.

## Supplementary Material

ofad449_Supplementary_Data

## Supplementary Data

Supplementary materials are available at *Open Forum Infectious Diseases* online. Consisting of data provided by the authors to benefit the reader, the posted materials are not copyedited and are the sole responsibility of the authors, so questions or comments should be addressed to the corresponding author.
