# Supplementary material for: Baseline Cytomegalovirus Viremia at Cryptococcal Meningitis Diagnosis Is Associated With Long-term Increased Incident TB Disease and Mortality in a Prospective Cohort of Ugandan Adults With HIV
Source: Open Forum Infect Dis. 2023 Sep 19;10(9):ofad449. doi: 10.1093/ofid/ofad449 (PMC10508356; doi:10.1093/ofid/ofad449)
Supplement: ofad449_Supplementary_Data [file ofad449_supplementary_data.docx]

| Supplementary table 1: Baseline characteristics of 497 two-week survivors of HIV-associated cryptococcal meningitis with stratification by receipt of CMV viral load testing | | | | |
| --- | --- | --- | --- | --- |
| Variable | **Total cohort**  **N (%) or median (IQR)** | **CMV viral load testing performed**  **N (%) or median (IQR)** | **No CMV viral load testing performed**  **N (%) or median (IQR)** | **P value^1^** |
| Total | 497 | 259 (52.1) | 238 (48.9) |  |
| Male | 297 (59.8) | 161 (54.2) | 136 (45.8) |  |
| Female | 200 (40.2) | 98 (49.0) | 102 (51.0) | 0.25 |
| Age (years) |  |  |  |  |
| *≤ 30* | 161 (32.4) | 83 (51.5) | 78 (48.5) |  |
| *31-50* | 320 (64.4) | 168 (52.5) | 152 (47.5) |  |
| *>50* | 16 (3.2) | 8 (50.0) | 8 (50.0) | 0.97 |
| ART |  |  |  |  |
| *No* | 322 (64.8) | 87 (49.7) | 88 (50.3) |  |
| *Yes* | 175 (35.2) | 172 (53.4) | 150 (46.6) | 0.43 |
| Median time on ART (days) | 167 (41-980) | 195 (41 – 1,076) | 163 (39 – 836) | 0.92 |
| CD4 category (cells/μL) |  |  |  |  |
| *< 50* | 340 (72.6) | 191 (56.2) | 149 (43.8) |  |
| *50-99* | 82 (17.5) | 46 (56.1) | 36 (43.9) |  |
| *≥100* | 46 (9.8) | 20 (43.5) | 26 (56.5) | 0.26 |
| HIV viral load (copies/ml) | 102,515 (1,580 -356,878) | 78,451 (260 - 360,976) | 167,725 (8,384 – 341,725) | 0.13 |
| GCS < 15 | 154 (31.1) | 74 (48.0) | 80 (52.0) |  |
| GCS = 15 | 341 (68.9) | 185 (54.2) | 156 (45.8) | 0.20 |
| CSF WCC (cells/μL) | 4 (4 - 80) | 4 (4 – 80) | 4 (4 – 75) | 0.94 |
| CSF QCC (cfu/ml) | 19,100 (500 – 209,000) | 35,000 (1,260-229,000) | 11,900 (200 -172,000) | 0.001 |
| History of TB | 94 (18.9) | 49 (52.1) | 45 (47.9) |  |
| No history of TB | 403 (81.1) | 210 (52.1) | 193 (47.9) | 0.99 |
| No TPT receipt | 381(76.7) | 196 (51.4) | 185 (48.6) |  |
| TPT receipt | 116 (23.3) | 63 (44.3) | 53 (45.7) | 0.59 |
| No Incident TB | 399 (80.3) | 209 (52.4) | 190 (47.6) |  |
| Incident TB | 98 (19.7) | 50 (51.0) | 48 (49.0) | 0.81 |
| Alive | 355 (71.4) | 188 (53.0) | 167 (47.0) |  |
| Died | 142 (28.6) | 71 (50.0) | 71 (50.0) | 0.55 |
| 1 = P-values compare participants with and without CMV viral load testing.  For categorical variables, the P value is from Chi squared tests.  For non-categorical variables p-values are from Mann-Whitney U tests throughout due to the non-parametric distribution of all data.  CMV = Cytomegalovirus, ART = anti-retroviral therapy, GCS = Glasgow Coma Scale, CSF = Cerebrospinal fluid, WCC = white cell count, QCC = quantitative cryptococcal culture. | | | | |

| Supplementary table 2: Mortality risk amongst 497 two-week survivors of HIV-associated cryptococcal meningitis stratified by clinical and laboratory baseline characteristics using univariate Cox regression analysis. | | | | |
| --- | --- | --- | --- | --- |
| Variable | **N** | **Death during study period N (%)** | **cHR**  **(95% CI)** | **P-value^1^** |
| Total | 497 | 142 (28.6) |  |  |
| Male | 297 | 90 (30.3) | 1 |  |
| Female | 200 | 52 (26.0) | 0.85 (0.61-1.20) | 0.37 |
| Age (years) |  |  |  |  |
| *≤ 30* | 161 | 51 (31.7) | 1 |  |
| *31-50* | 320 | 85 (26.6) | 0.66 (0.28-1.54) |  |
| *>50* | 16 | 6 (37.5) | 0.54 (0.23-1.23) | 0.24 |
| ART |  |  |  |  |
| *Yes* | 175 | 43 (24.6) | 1 |  |
| *No* | 322 | 99 (30.7) | 1.08 (0.75-1.56) | 0.66 |
| CD4 category (cells/μL) |  |  |  |  |
| *< 50* | 340 | 103 (30.3) | 1 |  |
| *50-99* | 82 | 16 (19.5) | 0.64 (0.38-1.09) |  |
| *≥100* | 46 | 12 (26.1) | 0.85 (0.47-1.55) | 0.21 |
| GCS < 15 | 154 | 43 (27.9) | 1 |  |
| GCS = 15 | 341 | 98 (28.7) | 1.01 (0.71-1.45) | 0.95 |
| No history of TB | 403 | 114 (28.3) | 1 |  |
| History of TB | 94 | 28 (29.8) | 1.05 (0.69-1.59) | 0.81 |
| No TPT receipt | 381 | 141 (37.0) | 1 |  |
| Receipt of TPT | 116 | 1 (0.86) | 0.01 (0.001-0.78) | <0.001 |
| 1 = P-values from Cox Regression analysis Wald test  CMV = Cytomegalovirus  ART = anti-retroviral therapy  GCS = Glasgow Coma Scale  TPT = TB preventive therapy  cHR = crude Hazard Ratio | | | | |

| Supplementary table 3: Baseline characteristics of 259 adults with HIV-associated cryptococcal meningitis with stratification by CMV viral load at baseline | | | | |
| --- | --- | --- | --- | --- |
| Variable | **Total cohort with CMV VL**  **N (%) or median (IQR)** | **No CMV viraemia / viraemia <1000 IU/ml**  **N (%) or median (IQR)** | **CMV viraemia ≥1000 CMV IU/ml**  **N (%) or median (IQR)** | **P value^1^** |
| Total | 259 | 223 (86.1) | 36 (13.9) |  |
| Male | 161 (62.2) | 138 (85.7) | 23 (14.3) |  |
| Female | 98 (37.8) | 85 (86.7) | 13 (13.3) | 0.82 |
| Age (years) |  |  |  |  |
| *≤ 30* | 83 (32.0) | 69 (83.1) | 14 (16.9) |  |
| *31-50* | 168 (64.9) | 146 (86.9) | 22 (13.1) |  |
| *>50* | 8 (3.1) | 8 (100) | 0 (0) | 0.37 |
| ART |  |  |  |  |
| *No* | 172 (66.4) | 143 (83.1) | 29 (16.9) |  |
| *Yes* | 87 (33.6) | 80 (91.9) | 7 (8.1) | 0.05 |
| Median time on ART (days) | 195 (41 – 1,076) | 166 (38 – 938) | 1,187 (43 – 2,270) | 0.29 |
| CD4 category (cells/μL) |  |  |  |  |
| *< 50* | 191 (74.3) | 159 (83.2) | 32 (16.8) |  |
| *50-99* | 46 (17.9) | 43 (93.5) | 3 (6.5) |  |
| *≥100* | 20 (7.8) | 19 (95.0) | 1 (5.0) | 0.10 |
| Median HIV viral load  (copies/ml) | 126,725  (8,384 – 341,919) | 112,088  (4,111 – 337,150) | 207,202  (74,383 – 387,068) | 0.17 |
| GCS < 15 | 74 (28.6) | 61 (82.4) | 13 (17.6) |  |
| GCS = 15 | 185 (71.4) | 162 (87.6) | 23 (12.4) | 0.28 |
| Median CSF WCC (cells/μL) | 4 (4 – 80) | 4 (4 – 90) | 4 (4 – 25) | 0.02 |
| Median CSF protein (mg/dL) | 60 (27-124) | 60 (27-124) | 50 (24-88) | 0.43 |
| Median CSF QCC (cfu/ml) | 35,000  (1,260 – 229,000) | 19,200  (710 – 214,000) | 112,500  (29,850 – 456,500) | 0.006 |
| History of TB | 210 (81.1) | 42 (85.7) | 7 (14.3) | 0.93 |
| Receipt of TPT | 49 (18.9) | 57 (90.5) | 6 (9.5) | 0.25 |
| 1 = P-value compares those without CMV viraemia or CMV viraemia <1000IU/ml) vs. those with CMV viraemia ≥1000 CMV IU/ml at time of cryptococcal meningitis diagnosis.  For categorical variables, the P value is from Chi squared if there were ≥5 participants in each category, or from Fisher’s exact if <5 participants. For non-categorical variables p-values are from Mann-Whitney U tests throughout due to the non-parametric distribution of all data.  CMV = Cytomegalovirus, VL = Viral load, ART = anti-retroviral therapy, GCS = Glasgow Coma Scale, CSF = Cerebrospinal fluid, WCC = white cell count, QCC = quantitative cryptococcal culture. | | | | |
